# Correlation between p53 immunoexpression and *TP53* mutation status in extrapulmonary small cell neuroendocrine carcinomas and its association with patient survival

**DOI:** 10.1007/s00428-025-04024-6

**Published:** 2025-01-23

**Authors:** Klára Pavlíčková, Jan Hojný, Petr Waldauf, Pavel Dundr, Nikola Hájková, Marián Švajdler, Pavel Fabian, Iva Staniczková Zambo, Miroslava Flídrová, Jan Laco, Helena Hornychová, Patricie Delongová, Jozef Škarda, Jan Hrudka, Radoslav Matěj

**Affiliations:** 1 Department of Pathology and Molecular Medicine, Thomayer University Hospital, Third Faculty of Medicine, Charles University, Prague, Czech Republic; 2 Department of Pathology, First Faculty of Medicine, Charles University and General University Hospital in , Prague, Prague, Czech Republic; 3 Department of Anaesthesia and Intensive Care, Third Faculty of Medicine, Charles University and University Hospital Kralovske Vinohrady, Prague, Czech Republic; 4 Bioptická laboratoř S.R.O. and Šikl’s, Department of Pathology, Charles University, Medical Faculty in Pilsen, Pilsen, Czech Republic; 5 Department of Oncological Pathology, Masaryk Memorial Cancer Institute, Brno, Czech Republic; 6 1, st Institute of Pathologic Anatomy, St. Anne’s University Hospital and Faculty of Medicine, Masaryk University, Brno, Czech Republic; 7 The Fingerland Department of Pathology, Charles University, Faculty of Medicine in Hradec Králové and University Hospital Hradec Kralove, Hradec Králové, Czech Republic; 8 Department of Clinical and Molecular Pathology and Medical Genetics, University Hospital Ostrava and Faculty of Medicine, University of Ostrava, Ostrava, Czech Republic; 9 Department of Pathology, University Hospital Kralovske Vinohrady, Third Faculty of Medicine, Charles University, Prague, Czech Republic

**Keywords:** Extrapulmonary small cell neuroendocrine carcinoma, *TP53* molecular analysis, p53 immunohistochemical analysis, Next generation sequencing

## Abstract

Extrapulmonary small cell neuroendocrine carcinoma (EP-SCNC) is a rare malignancy with a poor prognosis. Despite its morphological similarity to lung small cell carcinomas, its oncogenesis remains uncertain. One hundred and seventy-one EP-SCNC were enrolled in a multicenter study, and all tissue samples underwent an immunohistochemical p53 analysis. One hundred twenty-five samples were molecularly analyzed using next-generation sequencing (NGS), comprising DNA and RNA analysis. p53 normal/wild type expression was detected in 68 cases (39.8%), whereas aberrant expression was detected in 103 cases (60.2%). Molecular *TP53* alteration was detected in 92 out of 125 tumors (73.6%). The *TP53* mutation was shown to be prognostic and associated with shorter overall survival (*p* = 0.041). The multivariate analysis of p53 and *TP53* mutational status found that it impacted overall survival relative to distinct sites of tumor locations (*p* = 0.004 and *p* = 0.001, respectively). Age did not influenced survival in the multivariate analysis of p53 and *TP53* (*p* = 0.002; *p* < 0.001 resp.). Among tumors with paired immunohistochemical and molecular results, 108 exhibited concordance between the immunohistochemical and molecular analysis, whereas 17 were discordant. Accordingly, p53 aberrant expression was tightly associated with a *TP53* mutation (*p* < 0.001). In discordant cases, molecular analysis revealed no alteration in three tumors with p53 overexpression. In contrast, in 14 tumors with wild-type p53 expression, *TP53* genetic alteration was detected. Possible causes of discordance are discussed in this manuscript. Furthermore, the incidence of aberrant p53 expression / *TP53* molecular alteration was noticeably lower in EP-SCNC than in small-cell lung carcinomas. Therefore, in EP-SCNC, other driver mutations should be sought since personalized therapy can improve patient prognosis.

**Supplementary Information:**

The online version contains supplementary material available at 10.1007/s00428-025-04024-6.

## Introduction

The *TP53* gene, localized on chromosome 17p13.1, encodes tumor suppressor protein p53. Alterations in *TP53* represent the most common molecular abnormalities in human oncogenesis, with more than half of malignancies exhibiting *TP53* mutations [1].

Normal p53 protein is a transcription factor involved in the inhibition of cell cycle progression, induction of apoptosis, and promotion of senescence [2].

In the genetic alteration of *TP53*, there is abnormal protein accumulation as a result of altered stabilization of the protein [3], which can be detected via immunohistochemistry (IHC). p53 immunoexpression is, therefore, considered to be a relevant predictor of an underlying *TP53* mutation. In contrast to *TP53* mutational analysis, IHC is easy to perform, quick, and inexpensive.

The p53 IHC scoring system varies in different tumor types. There are four types of p53 immunoexpression – (1) normal (wild type) expression and (2) abnormal (aberrant, mutation-type) expression, where p53 can be either (2a) overexpressed, (2b) completely absent, or (2c) expressed in the cytoplasm of the cell. In wild-type p53 immunoexpression, variable nuclear expression, either weak or strong, is detected in less than 75% of tumor cells. p53 overexpression means strong nuclear positivity in at least 80% of the tumor cells. In completely negative tumors (with positive internal control staining), the p53 immunostaining is aberrant, i.e., completely absent.

In general, in-frame mutations lead to p53 overexpression. Disruption of genes (truncation, splice site mutations, deletions, frameshifts) is associated with the null type p53 immunophenotype [1, 4, 5].

Mutations in the *TP53* gene can lead to various conditions, described as “loss-of-function,” “dominant negative,” and “gain-of-function.” In “loss-of-function,” p53 does not maintain its tumor-suppressor function. “Dominant-negative” is represented by wild-type p53 inhibition by mutated p53 protein (originating from one mutant allele). In “gain-of-function,” p53 behaves similar to an oncogene [6].

The type of genetic alteration further defines p53 functionality [2] – for example, some *TP53* mutations can lead to preserved ability to arrest cell cycles but lose the ability to induce apoptosis at the same time or vice versa, i.e., a *TP53* mutation can lead to retained ability to induce programmed cell death while losing the ability to control the cell cycle.

Many advances in our understanding of *TP53* and p53 biology came from early attempts to target the p53 protein as a part of cancer therapy.

Extrapulmonary small cell neuroendocrine carcinoma (EP-SCNC) is a rare malignancy with a poor prognosis [7]. EP-SCNC is thought to arise from a multipotent stem cell. However, molecular evidence has recently suggested that EP-SCNC can develop late in the progression of various types of organ-specific carcinomas [7]. Moreover, the incidence of extrapulmonary neuroendocrine carcinoma is increasing, which may be related to expanded systemic cancer therapy for organ-specific adenocarcinomas [8]. Therefore, the molecular alterations in EP-SCNC may differ from those described in lung small cell carcinoma (LSCC), where the *TP53* mutation is a well-known and frequent genetic alteration [9].

This study aimed to complete an immunohistochemical p53 and molecular *TP53* analysis in a large cohort of EP-SCNC patients and correlate p53/*TP53* status with patient survival.

## Materials and methods

### Sample selection

The original study included 171 samples of diagnosed EP-SCNC over 24 years (from 1999 to 2023). Small cell carcinomas with a neuroendocrine morphology (high-grade tumors consisting of tumor cells with scant cytoplasm, finely granular chromatin without nucleoli, brisque mitotic activity, frequent apoptotic bodies, and necroses) but without known pulmonary involvement were included, regardless of expression of neuroendocrine markers although expression of three standard markers (i.e., INSM1, synaptophysin, and chromogranin A) was analyzed in all samples. Other types of tumors were carefully excluded using immunohistochemistry at the time of diagnosis. The study was multicentered, with specimens from the archives of all participating pathology departments.

Formalin-fixed, paraffin-embedded (FFPE) tissue from biopsies or surgical resection were collected. Hematoxylin and eosin-stained slides were reviewed by two pathologists (R.M. and K.P.). Representative areas were selected from the FFPE blocks, and tissue microarrays (TMA) were constructed. Other representative parts of tumors were selected for next-generation sequencing (NGS); the percentage of vital cells was estimated semi-quantitatively.

Clinical data included sex, age at diagnosis, and primary origin of the tumor. Overall survival (OS), i.e., the time between the initial diagnosis and death, came from the Institute of Health Information and Statistics of the Czech Republic. The study was performed with the approval of the Committee for Ethics in Science, Czech Academy of Science.

### Immunohistochemical analysis

IHC was performed on TMAs using four μm thick sections of FFPE tissue. For the construction of the TMAs, eligible areas of each tumor were identified, and two tissue cores (each 2.0 mm in diameter) were taken from the donor block using the tissue microarray instrument TMA Master (3DHISTECH Ltd., Budapest, Hungary). Using TMAs, IHC was performed for p53, using the DO-7 antibody (DakoCytomation, mouse, dilution 1:70). In discordant cases (IHC aberrant staining and non-mutated *TP53*, or IHC wild type staining and mutated *TP53*), IHC was re-done on the whole tissue section.

p53 immunohistochemical expression was scored as wildtype, aberrant–null expression, aberrant with cytoplasmic expression, or aberrant–overexpressed, i.e., in cases where more than 75% of the tumor cells displayed strong nuclear positivity (described in detail elsewhere) [4].

In ambiguous cases, both pathologists (R.M. and K.P.) re-evaluated the slides together to reach a consensus.

### Molecular analysis of TP53 mutation status

DNA for NGS was isolated from FFPE tissue sections using Quick-DNA/RNA FFPE Miniprep Kits (Zymo Research) according to the manufacturer’s instructions. NGS libraries were prepared using KAPA Hyper Plus kits (Roche) and HyperCapture (Roche) targeted custom panels (786 genes or gene parts; 2440 kbp of the target sequence; 1992 kbp of coding sequence), which included coding sequences for all *TP53* exons with ± 20 bp flanking intronic areas. Enriched libraries were sequenced using an Illumina NovaSeq 6000. Details of library preparation, sequencing, bioinformatic analysis, and data interpretation were previously described [10]. The damaging effect of detected variants was evaluated using the OncoKB [11] and/or ClinVar [12] databases.

In our study, if the tumor content in the FFPE specimen was < 40%, a microdissection was performed to augment tumor cell proportions. At the same time, in combined tumors, tissue microdissection was used to obtain only the small cell carcinoma constituent.

## Statistical analysis

All analyses were performed using R version 4.4.1 and RStudio; survival analyses were carried out using the survival package 3.7), survminer 0.4.9 and adjustedCurves 0.11.2 [13, 14]. Continuous data are expressed as means and standard deviations or medians with 25th and 75th percentiles (or interquartile range, IQR) depending on the normality of the distribution. Binary and categorical data are expressed as counts and percentages.

For OS analysis, a Kaplan–Meier analysis was performed with 95% confidence intervals (95% CIs) calculated using the log–log method and the p-value from the log-rank test. Univariate and age-adjusted Cox regressions were also performed for each parameter and expressed as unadjusted and age-adjusted hazard ratios (HR) with 95% CI. For OS analysis, patients were censored at five years.

The Pearson chi-square test and Fisher's exact test were used as appropriate to assess associations between categorized variables.

For p53 and *TP53*, a multivariable Cox regression analysis was performed, adjusted for the sex, age and tumor location. The results of the multivariable Cox regression are presented as adjusted hazard ratios (HR) with 95% confidence intervals (CI).

Cohen´s kappa, sensitivity, and specificity of immunohistochemical analysis and molecular analysis were calculated with regard to the *TP53* molecular analysis as a the most specific analysis, being a “gold standard”.

*P* values < 0.05 were considered statistically significant.

## Results

### Clinical data

Of the 171 patients included in the analysis, there were 103 males (60.6%) and 67 females (39.4%), with a mean age of 67 years (range 38.2–94.1 yrs.) at the time of diagnosis. Median survival was 9.36 months (95% CI 7; 12.3 months), five-year survival was 22%. All patients were of Caucasian origin. The primary EP-SCNC tumor location was the genitourinary tract in 81 patients (47.4%), gastrointestinal tract in 28 patients (16.4%), gynecological tract in 20 patients (11.7%), pancreas in nine patients (5.3%), head and neck in eight patients (4.7%), breast in six patients (3.5%), hepatobiliary system in six patients (3.5%), and the skin in one patient (0.6%). There were 12 EP-SCNC with an unknown primary site (7%) – in these tumors, as well as in all selected cases, a primary pulmonary origin was carefully and completely excluded. All specimens were from tumor resections/biopsies of primary tumors, apart from the 12 EP-SCNCs with an unknown primary site.

There were 98 biopsies and 73 resection specimens.

One hundred and forty-seven tumors (86%) were pure SCNC, whereas 24 tumors (14%) were combined with SCNC. Combined SCNC included acinar adenocarcinoma of the prostate and urothelial carcinoma, mixed colorectal adenocarcinoma, mixed ductal pancreatic adenocarcinoma, squamous cell carcinoma, and clear cell renal cell carcinoma.

Two patients were lost to follow-up for survival and expression analysis.

### Immunohistochemical analysis

P53 IHC was performed in 171 patients. Among them, p53 normal/wild type expression was detected in 68 cases (39.8%), overexpression was detected in 62 cases (36.3%), a complete absence of p53 staining was detected in 37 cases (21.6%), p53 cytoplasmic expression in one case (0.6%), and p53 overexpression with cytoplasmic expression at the same time was detected in three tumors (1.8%).

There was no difference in the proportion of wild-type/aberrant p53 expression relative to tumor locations (*p* = 0.093).

There was no difference in p53 expression in combined versus pure EP-SCNC (*p* = 0.3).

INSM1, synaptophysin, or chromogranin A immunoexpression was detected in 144 tumors (85.2%); 25 tumors were negative for all three markers. Neuroendocrine marker expression did not differ in tumors with wild-type or abnormal p53 expression (HR = 0.56, 95%CI 0.19, 1.52, *p* = 0.3).

### Molecular analysis

Molecular *TP53* alteration was detected in 92 out of 125 tumors (73.6%). In 90, a class 4/5 mutation was observed. In two tumors with p53 overexpression detected via IHC, three class three variants (variants with uncertain significance / inconclusive variants) were detected: NM_000546.5:c.1001G > T (p.Gly334Val) in sample #17 and NM_000546.5:c.656C > G (p.Pro219Arg) with NM_000546.5:c.634 T > G (p.Phe212Val) in sample #170.

Complete NGS data are provided in Pavlickova et al. [15].

Forty-eight tumors had missense mutations, 23 tumors had truncational mutations, nine tumors had truncational mutations and missense mutations at the same time, five tumors had missense mutations and deletions at the same time, two tumors had missense mutations on both alleles, two tumors had truncational mutations on both alleles, and one tumor probably had a heterozygous *TP53* deletion (and wild type p53 immunoexpression).

### Immunohistochemical and molecular biological results correlation

Both immunohistochemical and molecular analysis was performed on 125 tumors. Among them, p53 normal/wild-type expression was detected in 44 cases (35.2%). In contrast, complete absence was found in 20 cases (16%), p53 overexpression was detected in 57 cases (45.6%), pure cytoplasmic expression was detected in one tumor (0.8%), and p53 overexpression with cytoplasmic expression at the same time was detected in three tumors (2.4%).

Concerning associations between immunohistochemical and molecular analysis, p53 aberrant expression was tightly associated with a *TP53* mutation (*p* < 0.001). Cohen´s kappa between the two methods was 0.618 (z = 6.96; p≺ 0.001). With regard to the *TP53* molecular analysis as a gold standard, the sensitivity and specificity of immunohistochemical analysis were 0.84 (95% CI 0.75; 0.91) and 0.81 (95% CI 0.65; 0.92), respectively.

Among tumors with paired IHC and molecular results, 108 exhibited concordance between IHC and molecular analysis, whereas 17 were discordant.

**Fig. 1:**
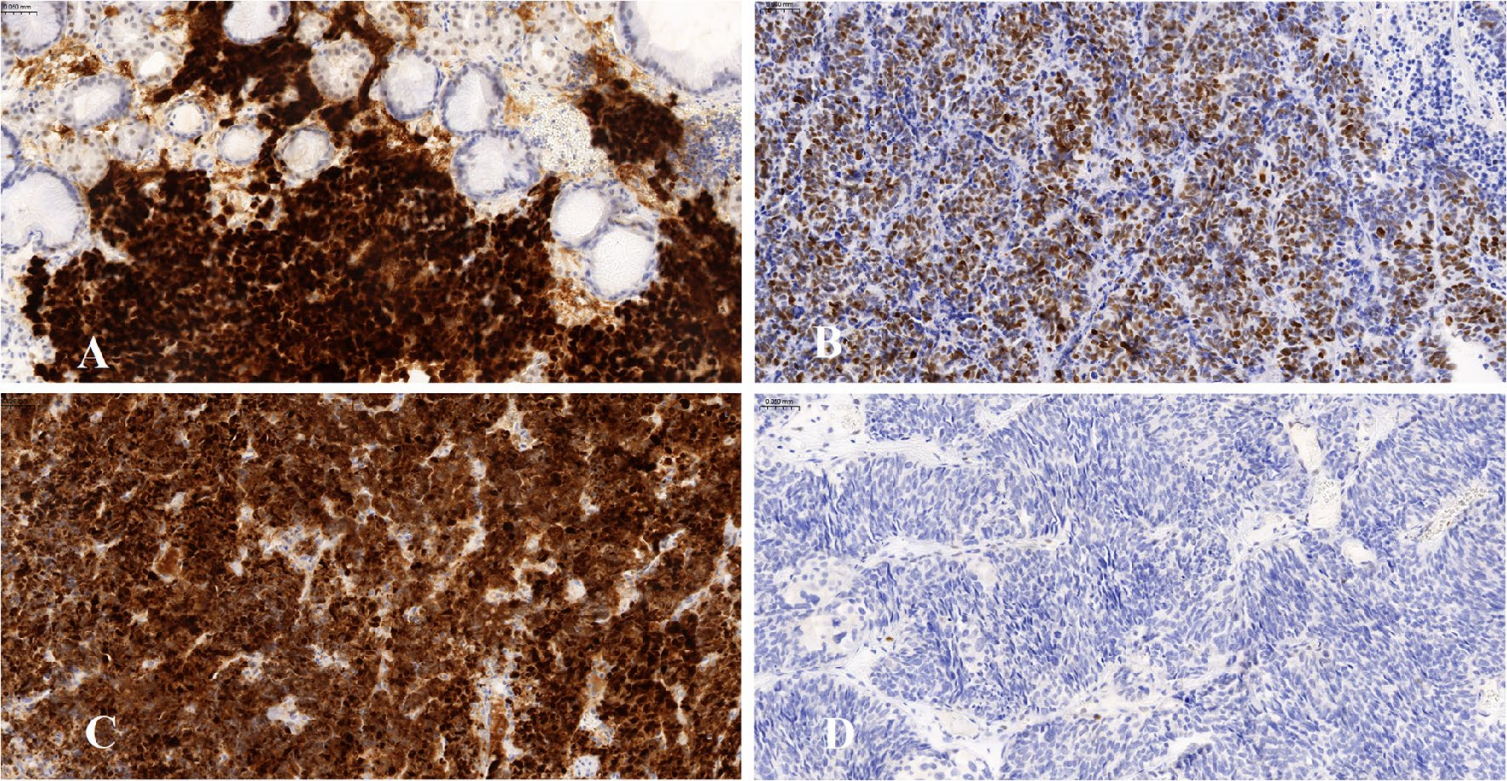
**A** Discordant case: EP-SCNC of stomach exhibiting p53 overexpression, whereas *TP53* alteration was not detected via NGS. 200 ×. **B** Discordant case: EP-SCNC of genitourinary tract with wild-type p53 immunoexpression and *TP53* alteration detected via NGS (NM_000546.5:c.833C > T (p.Pro278Leu)). 200 ×. **C** Concordant case exhibiting aberrant—cytoplasmatic p53 positivity and *TP53* mutation NM_000546.5:c.824G > A (p.Cys275Tyr). 200 ×. **D** Concordant case exhibiting aberrant – null p53 expression and *TP53* mutation via NGS NM_000546.5:c.200delC (p.Pro67fs). 200x

In discordant cases, molecular analysis revealed no alteration in three tumors with p53 overexpression (Fig. 1).

On the other hand, in 14 tumors with wild-type p53 expression (31.8%), *TP53* genetic alteration was detected (Fig. 1). Among these were seven missense mutations, one missense mutation with probably a heterozygous deletion, two truncational mutations, one probably a heterozygous deletion, and three truncational mutations with missense mutations.

Details for the tumors with discordant molecular analysis and protein expression analysis are illustrated in Table 1.

**Table 1:** Summary of p53 and *TP53* discordant cases

Sex	Age	Location	p53 IHC status	Type of mutation detected by NGS	*TP53* mutation nomenclature	Death	Combined tumors
female	74	GI tract	Wild type expression	M; D	NM_000546.5:c.374C > G (p.Thr125Arg)	1	0
female	67	Genitourinary	Wild type expression	M	NM_000546.5:c.380C > T (p.Ser127Phe)	1	0
male	73	Genitourinary	Wild type expression	M	NM_000546.5:c.833C > T (p.Pro278Leu)	1	1
female	86	Haed and neck	Wild type expression	M	NM_000546.5:c.742C > T (p.Arg248Trp)	1	0
male	70	Genitourinary	Wild type expression	T	NM_000546.5:c.772G > T (p.Glu258*)	1	0
male	66	Unknown primary	Wild type expression	M	NM_000546.5:c.817C > T (p.Arg273Cys)	1	0
male	70	Genitourinary	Wild type expression	M	NM_000546.5:c.856G > A (p.Glu286Lys)	1	0
male	69	Genitourinary	Wild type expression	T; M	NM_000546.5:c.993 + 1G > A (p.?); NM_000546.5:c.743G > A (p.Arg248Gln)	0	0
male	65	Unknown primary	Wild type expression	D		0	0
male	67	GI tract	Wild type expression	M	NM_000546.5:c.734G > A (p.Gly245Asp)	1	0
male	83	Haed and neck	Wild type expression	M	NM_000546.5:c.700 T > C (p.Tyr234His)	1	0
male	78	Genitourinary	Wild type expression	T	NM_000546.5:c.1024C > T (p.Arg342*)	1	1
male	61	Genitourinary	Wild type expression	T; M	NM_000546.5:c.740A > T (p.Asn247Ile); NM_000546.5:c.321C > G (p.Tyr107*)	1	0
male	74	Genitourinary	Wild type expression	T; M	NM_000546.5:c.659A > G (p.Tyr220Cys); NM_000546.5:c.993 + 1G > A (p.?)	0	0
male	68	GI tract	Over-expression	no mutation detected		1	0
female	38	Gynaecological	Over-expression	no mutation detected		0	0
female	45	Gynaecological	Over-expression	no mutation detected		0	0

*M* missense, *T* truncating, *VUS* variant of unknown significance, *H CNV* gene deletion

### Survival analysis

Median survival of the whole group was 9.4 months (95% CI 7.0; 12.3), and five-year survival was 22.5% (95% CI 12.0; 24.0%).

**Fig. 2:**
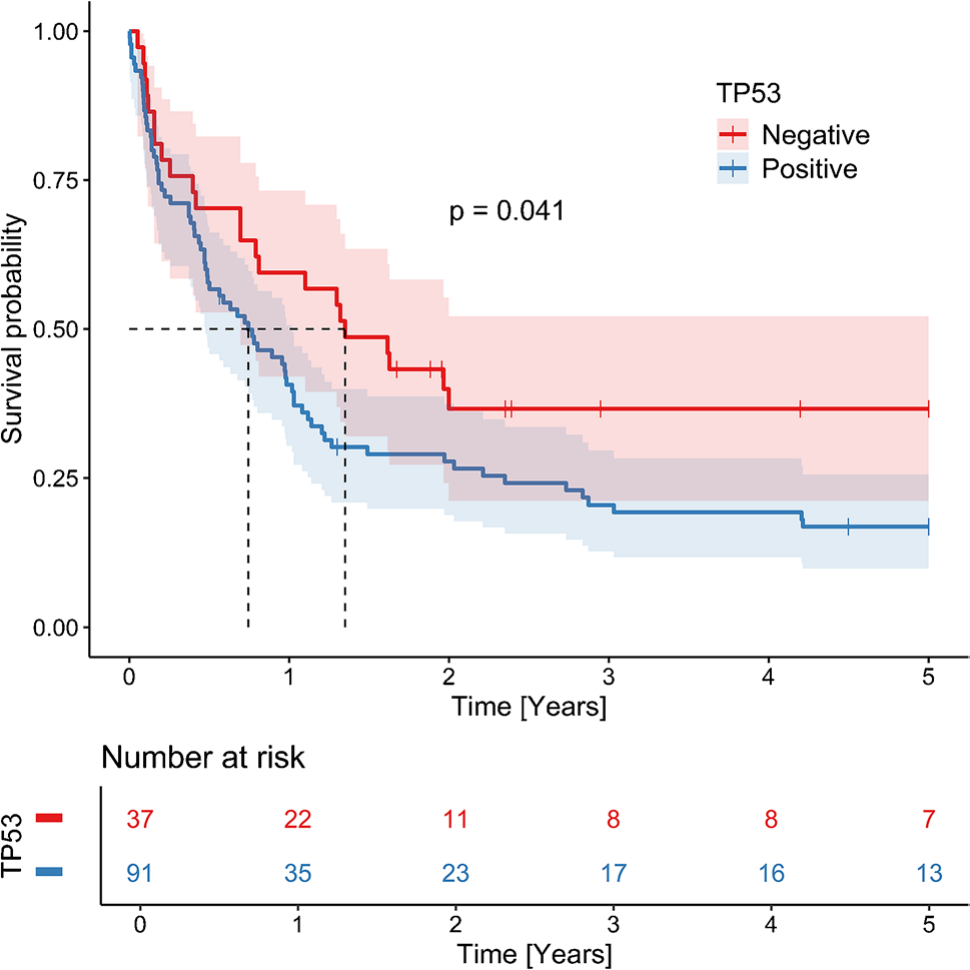
Kaplan–Meier curve documenting significantly shorter overall survival (OS) in patients with TP53 alteration

The presence of *TP53* mutation was shown to be a prognostic factor, associated with shorter OS (Kaplan–Meier analysis, median 8.9 months (95% CI 5.7; 12.2) vs. 16.2 months (95% CI 8.3; 62.6), *p* = 0.041. (Fig. 2).

In contrast, the results of p53 IHC did not have a statistically significant impact on OS (Kaplan–Meier analysis, 8.7 months, 95% CI (5.9;11.5) vs. 15.6 (8.2;23.7), *p* = 0.099.

To make our statistical analysis more robust, we performed a multivariate analysis that studied the prognostic impact of p53 immunoexpression and *TP53* mutational status relative to age and two distinct sets of tumor locations, i.e., the genitourinary and gynecological systems vs. the gastrointestinal system, the hepatobiliary system, and the pancreas.

Mutated *TP53* diagnosed by NGS remained significant predictor of worse survival if adjusted on patients age and tumour site (*n* = 110, HR = 1.84, 95%CI 1.09, 3.11; *p* = 0.023), similarly if examined by immunohistochemistry (*n* = 107, HR = 1.79, 95%CI 1.09, 2.86; *p* = 0.022). Gastrointestinal origo of the tumour was significant negative prognostic factor if adjusted on age and *TP53* NGS (*n* = 110, HR = 2.27, 95%CI 1.38, 3.71; *p* = 0.001) and immunohistochemistry (*n* = 107, HR = 2.07, 95%CI 1.27, 3.37; *p* = 0.004) status. Age did not influenced survival in the multivariate analysis (*n* = 110, HR = 1.03, 95%CI 1.01, 1.05; *p* = 0.002; *n* = 107, HR = 1.03, 95% CI 1.01, 1.06; *p* < 0.001 resp.).

Results of multivariate analysis are summarized in Supplementary Material 2.

## Discussion

*TP53* molecular alteration was detected in 39.8% EP-SCNC, whereas p53 aberrant immunoexpression was detected in 60.2% EP-SCNC. This is clearly a smaller proportion than in small cell lung carcinomas (SCLC), where up to 90% of tumors exhibit *TP53* mutations [16], which is therefore considered a driver mutation in the pathogenesis of SCLC. Our results are supported by previous studies, where EP-SCNC was shown to have a smaller proportion of *TP53* mutations [17, 18]. Many aspects of EP-SCNC oncogenesis still need to be clarified. EP-SCNC is known to develop from multipotent stem cells and can develop during the progression of various organ-specific carcinomas. Therefore, genetic alterations commonly seen in adenocarcinomas can also be detected in these tumors rather than *TP53* mutations [9, 19]. This is why we expected a smaller proportion of *TP53* mutations in combined EP-SCNC within our cohort, which was ultimately not finally demonstrated since 70.8% of combined EP-SCNC displayed aberrant-type p53 immunoexpression.

There was a strong association between p53 immunoexpression and *TP53* genetic alteration. Although in contrast to *TP53* mutational status, p53 immunoexpression did not have a prognostic impact in the univariate analysis; however, it was clearly shown in the multivariate analysis.

Even though SCLC and EP-SCNC are histologically and immunohistochemically identical tumors, they differ in tumorigenesis. Moreover, with our increasing knowledge of targeted therapy, targetable genetic alteration should be carefully sought in patients with EP-SCNC.

Currently, platinum-based chemotherapy is used to treat EP-SCNC as a standard treatment protocol. On progression, there is no consensus on second-line therapy [20, 21]. We may suppose more aggressive tumour behaviour based on p53 aberrant expression / *TP53* mutation, which, however, doesn´t have an impact on therapeutic strategy so far. However, besides its potential prognostic role, there have been first attempts in restoring the p53-pathway in various tumour types [22].

Concerning the types of *TP53* mutations, single-pair substitutions leading to the translation of a different amino acid, resulting in a full-length protein, is the most common genetic alteration in *TP53* gene [6]. This was supported by our study, since there were 64 missense mutations out of 92 *TP53* mutations detected.

Regarding p53/*TP53* discordant cases, numerous studies have reported discrepancies between p53 immunohistochemical and *TP53* molecular analysis across various tumors. Limitations in both methods, IHC and NGS, as well as tumor heterogeneity, may play a significant role in these discrepancies [23, 24].

IHC results can be modified or even blurred by preanalytical factors (e.g., poor tissue fixation). Due to the clonal heterogeneity of the tumor, TMA may complicate the analysis. In all discordant cases, we repeated IHC using the whole FFPE sample; however, the result of IHC was the same as in TMA. Additionally, correct interpretation of p53 IHC is essential. Both pathologists (R.M. and K.P.) re-evaluated the slides together to reach a consensus in uncertain cases. Despite this, we identified 14 “false positive” NGS results in cases with normal p53 expression, suggesting that tumor heterogeneity can play a significant role. Small cell neuroendocrine carcinoma is well known for having a high mutation rate [15, 25], therefore, intratumoral subpopulations are easily imaginable. Alternatively, some of the detected missense *TP53* variants could reduce p53 turnover in tumor cells without entirely disrupting the expression observed with IHC, as discussed by Boldrin et al. [24].

NGS results can also be affected by tissue quality and processing, e.g., small amounts of tissue as well as samples containing fragmented DNA, a necrotic tumor, or high non-tumor content [26, 27]. In our study, if the tumor content in the FFPE specimen was < 40%, a microdissection was performed to augment the tumor cell proportion. Another limiting factor could have been the technical limitations of our method, i.e., only coding sequences with flanking intronic regions of *TP53* were analyzed. In contrast, deep intronic or promoter regions were omitted.

Nevertheless, discordant results (NGS *TP53* without detected mutation, aberrant p53 expression) were only detected in three cases, with epigenetic changes of *TP53* being a potential explanation. *TP53* is typically not silenced by hypermethylation [28]; there are also negative regulators of p53 (for instance, Mdm2 and MdmX) [29]. However, none of these were detected in our discordant cases via NGS [15]. Moreover, several DNA viruses can inactivate p53 by altered protein stabilization [30].

Due to the improvement of our understanding of oncogenesis, the interpretation of NGS results can differ over time. In our study, two “variants of unknown significance” were referred after description of aberrant p53 immunoexpression. Although our observations in these cases cannot change the current classification of detected variants, they may serve as a basis for warranting a more thorough functional analysis of these variants.

Short follow-up in some patients (diagnosed between 1999–2023) may represent limitation in the statistical analysis.

## Conclusion

To our knowledge, ours is the first p53 immunoexpression / TP53 mutation study in a large cohort of neuroendocrine tumors with various primary locations*.* A *TP53* mutation proved to be a prognostic marker within our cohort. There was a statistical correlation between p53 immunoexpression and *TP53* genetic alteration. However, *TP53* mutations were detected to a lesser extent in EP-SCNC than in pulmonary SCC. Therefore, other driver mutations should be sought in cases of EP-SCNC, especially during this time of rapid development of targeted therapy [15].

## Supplementary Information

Below is the link to the electronic supplementary material.

Supplementary file1 Complete results on EP-SCNC p53 immunohistochemical and TP53 molecular analysis. Abbreviations: p53 IHC: 0: wild-type expression; 1: null expression; 2: overexpression; 3: overexpression and cytoplasmatic expression; 4: cytoplasmatic expression; TP53 molecular analysis: M: missense; T: truncating; VUS: variant of unknown significance; H: CNV gene deletion (XLSX 2610 KB)

Supplementary file2 Results on a multivariable Cox regression analysis, adjusted for the sex, age and tumor location (XLSX 11 KB)

## References

[1] Levine AJ, Oren M (2009) The first 30 years of p53: growing ever more complex. Nat Rev Cancer 9(10):749–758 19776744 10.1038/nrc2723 PMC2771725

[2] Prives C, Hall PA (1999) The p53 pathway. J Pathol 187(1):112–126 10341712 10.1002/(SICI)1096-9896(199901)187:1<112::AID-PATH250>3.0.CO;2-3

[3] Chan JK, Ip YT, Cheuk W (2013) The utility of immunohistochemistry for providing genetic information on tumors. Int J Surg Pathol 21(5):455–475 24065374 10.1177/1066896913502529

[4] Sung YN, Kim D, Kim J (2022) p53 immunostaining pattern is a useful surrogate marker for TP53 gene mutations. Diagn Pathol 17(1):92 36471402 10.1186/s13000-022-01273-w PMC9720942

[5] Jimbo N, Ohbayashi C, Fujii T, Takeda M, Mitsui S, Tsukamoto R, Tanaka Y, Itoh T, Maniwa Y (2023) Implication of cytoplasmic p53 expression in pulmonary neuroendocrine carcinoma using next-generation sequencing analysis. Histopathology; 84(2):336–342 10.1111/his.15059 37814580

[6] Hernandez Borrero LJ, El-Deiry WS (2021) Tumor suppressor p53: Biology, signaling pathways, and therapeutic targeting. Biochim Biophys Acta Rev Cancer 1876(1):188556 33932560 10.1016/j.bbcan.2021.188556 PMC8730328

[7] Frazier SR, Kaplan PA, Loy TS (2007) The pathology of extrapulmonary small cell carcinoma. Semin Oncol 34(1):30–38 17270663 10.1053/j.seminoncol.2006.11.017

[8] Stelwagen J, de Vries EGE, Walenkamp AME (2021) Current Treatment Strategies and Future Directions for Extrapulmonary Neuroendocrine Carcinomas: A Review. JAMA Oncol 7(5):759–770 33630040 10.1001/jamaoncol.2020.8072

[9] Sutherland KD, Proost N, Brouns I, Adriaensen D, Song JY, Berns A (2011) Cell of origin of small cell lung cancer: inactivation of Trp53 and Rb1 in distinct cell types of adult mouse lung. Cancer Cell 19(6):754–764 21665149 10.1016/j.ccr.2011.04.019

[10] Struzinska I, Hajkova N, Hojny J, Krkavcova E, Michalkova R, Bui QH, Matej R, Laco J, Drozenova J, Fabian P et al (2024) Somatic Genomic and Transcriptomic Characterization of Primary Ovarian Serous Borderline Tumors and Low-Grade Serous Carcinomas. J Mol Diagn; 26(4):257–266 10.1016/j.jmoldx.2023.12.004 38280423

[11] OncoKB. In*.*, https://www.oncokb.org/. Accessed 24 Nov 2023

[12] ClinVar. In*.*, database 20230819. https://www.ncbi.nlm.nih.gov/clinvar/. Accessed 24 Nov 2023

[13] Team RC (2024) A Language and Environment for Statistical Computing_. R Foundation for Statistical https://www.r-project.org/

[14] M TT (2024) A Package for Survival Analysis in R. R package version 3.7–0 https://cran.r-project.org/web/packages/survival/vignettes/survival.pdf

[15] Pavlíčková K, Hojny J, Waldauf P, Svajdler M, Dudr P, Fabian P et al Molecular and Immunohistochemical Classification of Extrapulmonary Small Cell Neuroendocrine Carcinomas: A Study of 181 Cases. Lab Invest in press. 10.1016/j.labinv.2025.104093 10.1016/j.labinv.2025.104093 39826683

[16] George J, Lim JS, Jang SJ, Cun Y, Ozretic L, Kong G, Leenders F, Lu X, Fernandez-Cuesta L, Bosco G et al (2015) Comprehensive genomic profiles of small cell lung cancer. Nature 524(7563):47–53 26168399 10.1038/nature14664 PMC4861069

[17] Basturk O, Tang L, Hruban RH, Adsay V, Yang Z, Krasinskas AM, Vakiani E, La Rosa S, Jang KT, Frankel WL et al (2014) Poorly differentiated neuroendocrine carcinomas of the pancreas: a clinicopathologic analysis of 44 cases. Am J Surg Pathol 38(4):437–447 24503751 10.1097/PAS.0000000000000169 PMC3977000

[18] Konukiewitz B, Schlitter AM, Jesinghaus M, Pfister D, Steiger K, Segler A, Agaimy A, Sipos B, Zamboni G, Weichert W et al (2017) Somatostatin receptor expression related to TP53 and RB1 alterations in pancreatic and extrapancreatic neuroendocrine neoplasms with a Ki67-index above 20. Mod Pathol 30(4):587–598 28059098 10.1038/modpathol.2016.217

[19] Yuan J, Knorr J, Altmannsberger M, Goeckenjan G, Ahr A, Scharl A, Strebhardt K (1999) Expression of p16 and lack of pRB in primary small cell lung cancer. J Pathol 189(3):358–362 10547597 10.1002/(SICI)1096-9896(199911)189:3<358::AID-PATH452>3.0.CO;2-1

[20] Stelwagen J, de Vries EGE, Walenkamp AME (2021) Current Treatment Strategies and Future Directions for Extrapulmonary Neuroendocrine Carcinomas: A Review. JAMA Oncol 7(5):759–770. 10.1001/jamaoncol.2020.8072 33630040 10.1001/jamaoncol.2020.8072

[21] McNamara MG, Frizziero M, Jacobs T et al (2020) Second-line treatment in patients with advanced extra-pulmonary poorly differentiated neuroendocrine carcinoma: a systematic review and meta-analysis. Ther Adv Med Oncol 12:1758835920915299. 10.1177/1758835920915299 32426044 10.1177/1758835920915299 PMC7222242

[22] Weinberg RA (1991) Tumor suppressor genes. Science 254(5035):1138–1146 1659741 10.1126/science.1659741

[23] Huang SC, Chang IY, Chen TC, Lin HC, Tsai CY, Hsu JT, Yeh CN, Chang SC, Yeh TS: Redefining aberrant P53 expression of gastric cancer and its distinct clinical significance among molecular-histologic subtypes. Asian J Surg 2024. 10.1016/j.asjsur.2024.05.121 38845323

[24] Boldrin E, Piano MA, Bernaudo F, Alfieri R, Biasin MR, Montagner IM, Volpato A, Mattara G, Lamacchia F, Magni G et al: p53/TP53 Status Assessment in Gastroesophageal Adenocarcinoma. Cancers (Basel) 2023, 15(10). 10.3390/cancers15102783 PMC10216170 37345120

[25] Su S, Zou JJ, Zeng YY, Cen WC, Zhou W, Liu Y, Su DH, Zhang XL, Huang HY, Lei A et al (2019) Tumor Mutational Burden and Genomic Alterations in Chinese Small Cell Lung Cancer Measured by Whole-Exome Sequencing. Biomed Res Int 2019:6096350 31781628 10.1155/2019/6096350 PMC6874933

[26] Meyerson M, Gabriel S, Getz G (2010) Advances in understanding cancer genomes through second-generation sequencing. Nat Rev Genet 11(10):685–696 20847746 10.1038/nrg2841

[27] Daniels M, Goh F, Wright CM, Sriram KB, Relan V, Clarke BE, Duhig EE, Bowman RV, Yang IA, Fong KM (2012) Whole genome sequencing for lung cancer. J Thorac Dis 4(2):155–163 22833821 10.3978/j.issn.2072-1439.2012.02.01 PMC3378223

[28] Kazanets A, Shorstova T, Hilmi K, Marques M, Witcher M (2016) Epigenetic silencing of tumor suppressor genes: Paradigms, puzzles, and potential. Biochim Biophys Acta 1865(2):275–288 27085853 10.1016/j.bbcan.2016.04.001

[29] Marine JC, Lozano G (2010) Mdm2-mediated ubiquitylation: p53 and beyond. Cell Death Differ 17(1):93–102 19498444 10.1038/cdd.2009.68

[30] Whang-Peng J, Kao-Shan CS, Lee EC, Bunn PA, Carney DN, Gazdar AF, Minna JD (1982) Specific chromosome defect associated with human small-cell lung cancer; deletion 3p(14–23). Science 215(4529):181–182 6274023 10.1126/science.6274023

